# Identification of HOXA1 as a Novel Biomarker in Prognosis of Head and Neck Squamous Cell Carcinoma

**DOI:** 10.3389/fmolb.2020.602068

**Published:** 2021-03-08

**Authors:** Hui Li, Xiaomin Wang, Mingjie Zhang, Mengjun Wang, Junjie Zhang, Shiyin Ma

**Affiliations:** Department of Otolaryngology, The First Affiliated Hospital of Bengbu Medical College, Bengbu, China

**Keywords:** hox genes, homeobox A1, head and neck squamous cell carcinoma, perineural invasion, prognosis, biomarker, tumor

## Abstract

Hox genes, a highly conserved homolog in most animals, play vital functions in cell development and organ formation. In recent years, researchers have discovered that it can act as a tumor regulator, and its members can participate in tumorigenesis by regulating receptor signaling, cell differentiation, apoptosis, migration, EMT, and angiogenesis. Hox genes and which major members play a vital role in the progress of head and neck squamous cell carcinoma (HNSCC) is still unclear. After analyzing the expression differences and prognostic value of all Hox genes through the TCGA-HNSC database, we use histochemistry stains in 52 pairs of HNSCC slices to verify the expression level of the key member-HOXA1. In correlation analysis, we found that high HOXA1 expression is related to poor pathological grade (*p* = 0.0077), advanced T stage (*p* = 0.021) and perineural invasion (PNI) (*p* = 0.0019). Furthermore, we used Cox univariate and multivariate regression analysis to confirm the independent predictive power of HOXA1 expression. To explore the underlying mechanisms behind HOXA1, we ran GSVA and GSEA and found fourteen mutual signaling pathways, including neuroprotein secretion and transport, tumor-associated signaling pathways, cell adhere junction and metabolic reprogramming. Finally, we found that the high expression of HOXA1 is significantly related to the decrease of CD8+ T cell infiltration and the decline of DNA methylation level. Our findings demonstrated that HOXA1, as a notable member of the HOX family, maybe an independent prognostic indicator in HNSCC.

## Introduction

Head and neck cancer was one of the most common malignant upper respiratory tract tumors globally, of which squamous cell carcinoma (HNSCC) accounts for about 90% (Bray et al., 2018). Although the continuous improvement of treatment such as surgery, radiotherapy, chemotherapy, targeted therapy and immunotherapy, the 5-year survival rate of HNSCC is still stagnant (Cancer Genome Atlas Network, 2015). Senile patients are mainly those who are habitually ingesting alcohol and tobacco, while HPV infection has led to an increase in young patients recently, especially oropharyngeal squamous cell carcinoma (Chaturvedi et al., 2011). Among them, patients with HPV-negative suffer from irreversible damage due to long-term smoking and drinking, who are more tolerant of the treatments with a worse prognosis.

Hox genes is a subgroup of the homeobox transcription factor family, which aggregates in 4 types of chromosomal loci (A-D), with 39 genes in humans. Hox gens plays its main function by encoding transcription factors or directly targeting downstream genes as monomers or homodimers (Alexander et al., 2009). Members of Hox genes can participate in cell development, organ formation and even carcinogenesis by regulating receptor signaling, cell differentiation, apoptosis, migration, epithelial-mesenchymal transition (EMT) and angiogenesis (Cho et al., 2012). Hox genes is tissue-specific and may play a key role in developmental regulation in one tissue type (Economides and Capecchi, 2003), while in another tissue, it is an accomplice of tumor malignant biological behavior (Wang et al., 2007). Previous studies have found that many Hox genes are abnormally expressed in hematological malignancies and various solid tumors. There are inadequate studies on the involvement of Hox genes in the occurrence and development of HNSCC. Therefore, studying the role of Hox genes in HNSCC is of great significance.

In this study, we used the method of bioinformatics mining database of TCGA-HNSC combined with immunohistochemical (IHC) verification to find a novel prognostic biomarker, HOXA1 (Homeobox A1) in Hox genes. HOXA1 is the first Hox gene to be discovered that can be upregulated during carcinogenesis by gastrin (Hofsli et al., 2005). In breast cancer, HOXA1 has been identified as a breast epithelial oncogene whose forced expression is sufficient to induce the transformation of immortalized human mammalian epithelial cells into aggressive cancer cells (Zhang et al., 2006; Mohankumar et al., 2008). In previous reports, the abnormal expression of HOXA1 is related to the prognosis of breast cancer, lung cancer, liver cancer, prostate cancer, oral cancer, gastric cancer and melanoma (Shah and Sukumar, 2010). We evaluated the abnormal expression of HOXA1 in HNSCC and confirmed its independent predictive power. At the same time, we analyzed the correlation between the expression level and clinicopathological characteristics. According to the expression level, we ran gene set variation analysis (GSVA) and gene set enrichment analysis (GSEA) to further analyze the biological pathways of HNSCC pathogenesis related to the expression of HOXA1. Finally, results of TIMER, CIBERSORT and MEXPRESS analysis indicated that HOXA1 was correlated with CD8+ T cell infiltration and DNA methylation level.

## Materials and Methods

### HOX Genes Expression Datasets and Relevant Clinicopathological Features

HNSCC dataset (528 cases) contain RNA-Seq (HTSeq) and clinicopathological characteristics from the cancer genome atlas (TCGA-HNSC) were downloaded (https://cancergenome.nih.gov/). Screened out 499 cases, including the expression of 39 Hox genes and corresponding clinicopathological features are used in the subsequent analysis. R Package “survminer” were used to calculate the cutoff value of Hox genes. The 499 cases were divided into a high-expression group (*n* = 396) and a low-expression group (*n* = 103) according to the cutoff value of HOXA1 expression (overall survival, OS: 1.0525, disease-free survival, DFS: 2.9749). Clinical features of HNSCC patients had been shown in Table 1.

**Table 1:** Clinical features of TCGA-HNSC patients (*n* = 499).

**Clinical characteristics**	**Total** **(*n* = 499)**	**Expression of HOXA1**		*p*-Value^^*^^
		**High**	**Low**	
		**(*n* = 396)**	**(*n* = 103)**	
Age, *n* (%)				0.8741
<65	311 (62.3)	248 (62.6)	63 (61.2)	
≥65	188 (37.7)	148 (37.4)	40 (38.8)	
Gender, *n* (%)				**0.0086**
Female	133 (26.7)	110 (27.8)	23 (22.3)	
Male	366 (73.3)	286 (72.2)	80 (77.7)	
Histologic grade, *n* (%)				**0.0023**
G1-2	362 (72.5)	300 (75.8)	62 (60.2)	
G3-4	121 (24.2)	84 (21.2)	37 (35.9)	
NA	16 (3.2)	12 (3.0)	4 (3.9)	
AJCC Stage, *n* (%)				1.0000
I-II	114 (22.8)	91 (23.0)	23 (22.3)	
III-IV	371 (74.3)	296 (74.7)	75 (72.8)	
NA	14 (2.8)	9 (2.3)	5 (4.9)	
T stage, *n* (%)				**0.0080**
T1-2	176 (35.3)	129 (32.6)	47 (45.6)	
T3-4	308 (61.7)	258 (65.2)	50 (48.5)	
NA	15 (3.0)	9 (2.3)	6 (5.8)	
N classification, *n* (%)				0.1761
N0 (negative)	238 (47.7)	197 (49.7)	41 (39.8)	
N1-3(positive)	239 (47.9)	185 (46.7)	54 (52.4)	
NA	22 (4.4)	14 (3.5)	8 (7.8)	
M classification, *n* (%)				1.0000
M0	469 (37.3)	374 (94.4)	95 (92.2)	
M1	5 (0.2)	4 (1.0)	1 (1.0)	
NA	25 (50.3)	18 (4.5)	7 (6.8)	
HPV_STATUS_P16, *n* (%)				1.0000
Positive	34 (6.8)	22 (5.6)	12 (11.7)	
Negative	78 (15.6)	61 (15.4)	17 (16.5)	
NA	387 (75.56)	313 (79.0)	74 (71.8)	
ANGIOLYMPHATIC_ INVASION, *n* (%)				0.5906
YES	218 (43.7)	92 (23.2)	28 (27.2)	
NO	120 (24.0)	174 (43.9)	44 (42.7)	
NA	161 (32.3)	130 (32.8)	31 (30.1)	
PERINEURAL_INVASION, *n* (%)				**0.00197**
YES	185 (37.1)	142 (35.9)	23 (22.3)	
NO	165 (33.1)	133 (33.6)	52 (50.5)	
NA	149 (29.8)	121 (30.6)	28 (27.2)	
Smoking pack / years, *n* (%)				0.5314
≥40	161 (32.3)	127 (32.1)	34 (33.0)	
<40	126 (25.3)	104 (26.3)	22 (21.4)	
NA	212 (42.4)	165 (41.7)	47 (45.6)	
ALCOHOL_HISTORY_ DOCUMENTED, *n* (%)				1.0000
YES	331 (66.3)	262 (66.2)	69 (67.0)	
NO	157 (31.5)	125 (31.5)	32 (31.1)	
NA	11 (2.2)	9 (2.3)	2 (1.9)	
Primary Tumor Site, *n* (%)				0.4934
Oropharynx	378 (75.8)	296 (74.7)	82 (79.6)	
Larynx	111 (22.2)	91 (23.0)	20 (19.4)	
Hypopharynx	10 (2.0)	9 (2.3)	1 (1.0)	

* *The chi-square test. Bold values indicate statistically significant, P < 0.05*.

### Differentially Expressed Genes and Clinical Pathological Parameters Analysis

First, the R package “limma” was used to analyze the differential expression of 39 Hox genes between tumor and normal samples in 499 patients and differentially expressed genes (DEGs) with |log2 fold change (FC)| > 1 and adjusted *P*-values < 0.05 were selected for subsequent analysis. The prognostic genes with the predictive ability of OS and DFS were screened out by the analysis of the Kaplan–Meier survival curve and the log-rank test (*P* < 0.05). The correlation analysis between the expression of HOXA1 and clinicopathological characteristics were evaluated by the R package “combn.” Furthermore, Cox univariate and multivariate regression analysis were performed on the individual clinical variables [age (years ≥65/ <65), gender (male/female), grade (G1&2/G3&4), AJCC Stage (I&II/III&IV), T stage (T1–2/T3–4), N classification (N0/N1–3), M classification (M0/M1), HPV_STATUS_P16 (positive/negative), angiolymphatic invasion (Yes/No), perineural invasion (Yes/No), smoking (packs/year ≥40/ <40), alcohol history documented (Yes/No) and primary tumor site (Oropharynx/ Larynx/Hypopharynx)]with the HOXA1 expression value and high or low-expression level. Hazard ratios (HRs) and 95% confidence intervals (CIs) were calculated using the R package “coxph.” The chi-square test was used to assess the statistical significance of expression value between the two groups.

### Analysis of Immune Infiltration and DNA Methylation

The fraction of twenty-two immune cell types of each TCGA-HNSC sample was yielded through R package “CIBERSORT” by estimating relevant subsets of RNA profiles (https://cibersort.stanford.edu/) (Supplementary Material 1). Only samples with *P* < 0.05 were conceived to perform the further analysis of comparing differential the tumor-infiltrating immune cells (TICs) level between the high and low-HOXA1 level group. In addition, another immune infiltration data is derived from TIMER (Tumor Immune Estimation Resource, https://cistrome.shinyapps.io/timer/) and DNA methylation data came from MEXPRESS database (https://mexpress.be/).

### Gene Set Variation Analysis (GSVA) and Gene Set Enrichment Analysis (GSEA)

The R package “GSVA” was used to evaluate the signaling pathways (5529 gene sets) and Gene Ontology (GO) terms (10,192 gene sets) in the TCGA-HNSC database (Hänzelmann et al., 2013). Then, the R package “limma” was used to analyze the significant differential gene sets between the two groups. Besides, 499 HNSCC cases were divided according to the median expression value of HOXA1. GSEA was then performed to detect the gene sets that were enriched in the gene rank for identifying potential the pathways most associated with HOXA1. The annotated gene sets of c2.cp.kegg. v7.1. symbols in the Molecular Signatures Database (MSigDB) was selected in GSEA version 4.1.0 (https://www.gsea-msigdb.org/gsea/msigdb/index.jsp). We performed 1,000 times of permutations and filtered the results by normalized enrichment scores (NES), nominal *p*-value and false discovery rate (FDR) q-value (Subramanian et al., 2005).

### Tissue Specimen Collection and Immunohistochemistry (IHC)

The study was approved by the Clinical Research Ethics Committee of The First Affiliated Hospital of Bengbu Medical College. Written informed consent was obtained from all-volunteer patients. In all, 52 pairs of HNSCC paraffin sections were collected from The First Affiliated Hospital of Bengbu Medical College (Supplementary Material 2). In the progress of antigen retrieval, the sections were placed in 10 mM citrate buffer solution (pH = 6.0) and boiling in a pressure cooker for 15 min. After cooling to 37°C, 3% H_2_O_2_ was added and incubated for 10 min to deplete the activity of endogenous peroxidase. After blocking by Goat serum, the sections were stained with an anti-HOXA1 polyclonal antibody (1:100, Thermo Fisher Scientific, catalog # PA5-36164, RRID AB_2553401) for a night at 4°C-refrigerator. After soaking and rinsing thoroughly with PBS three times, the sections were stained with secondary antibodies. Finally, the positive expression of the HOXA1 protein was observed by a microscope after adding substrate and hematoxylin staining. The expression density was scored by two experienced pathologists, following the single-blind trial technique. HOXA1 protein staining was assessed by the sum of the percentage of positive nuclear cells: − (0–25%), + (26–50%), ++ (51–100%). At last, we evaluated HOXA1 expression into two levels, low (− to +) and high (++). Fifty-two pairs of IHC pictures were taken for each slide, and images (200×) were analyzed using software ImageJ (US National Institute of Health, Bethesda, Maryland, USA, https://imagej.nih.gov/).

### Statistical Analysis

Statistical analyses were conducted with software R (v3.6.0) and GraphPad Prism 7 (GraphPad Software, California, USA). Differences among variables were analyzed with two-tailed Student's *t*-test and chi-square test. All significance tests were carried out at the 0.05 level.

## Results

### Differentially Expression and Prognostic Value of HOX Genes in HNSCC

**Figure 1:**
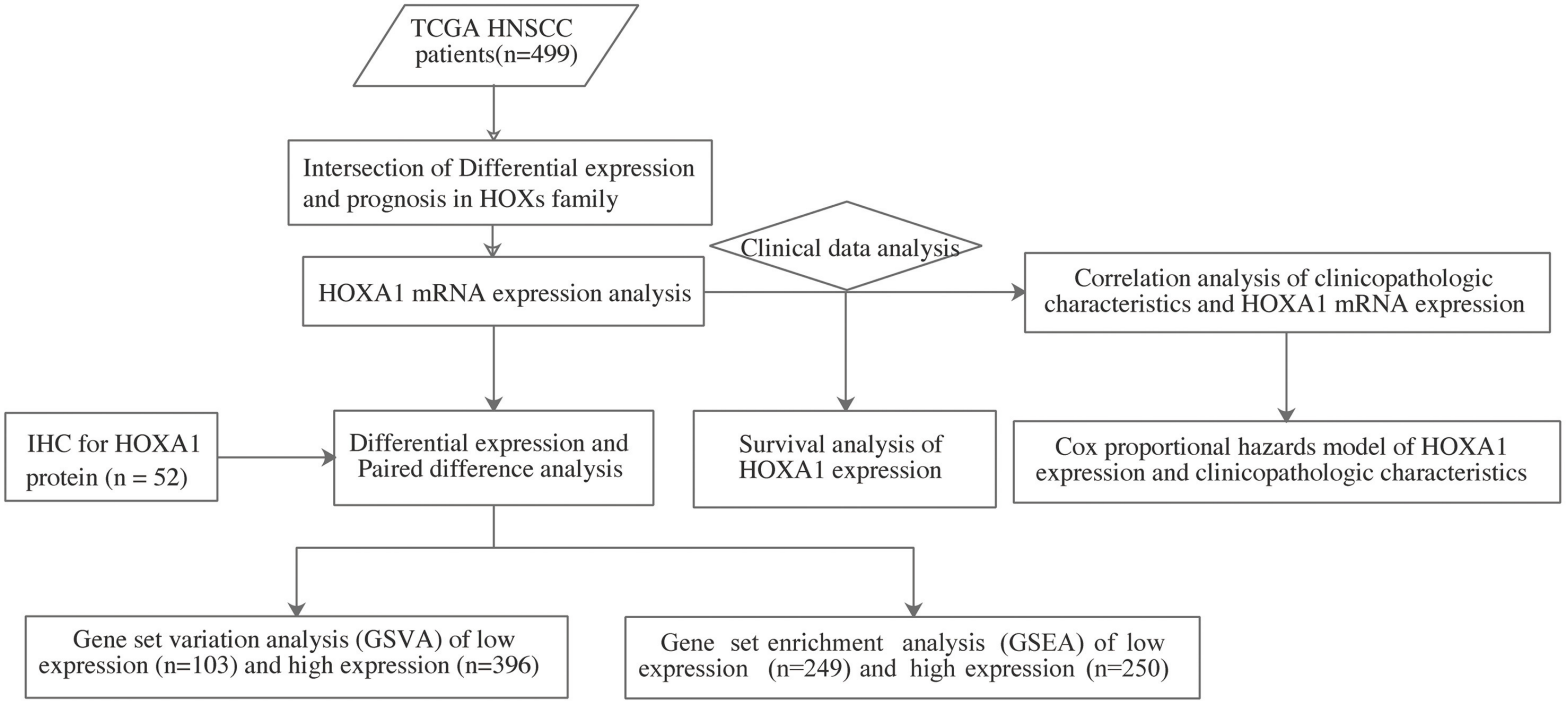
The flow chart showing the scheme of our study on HOXA1 as an independent prognostic indicator for HNSCC.

**Figure 2:**
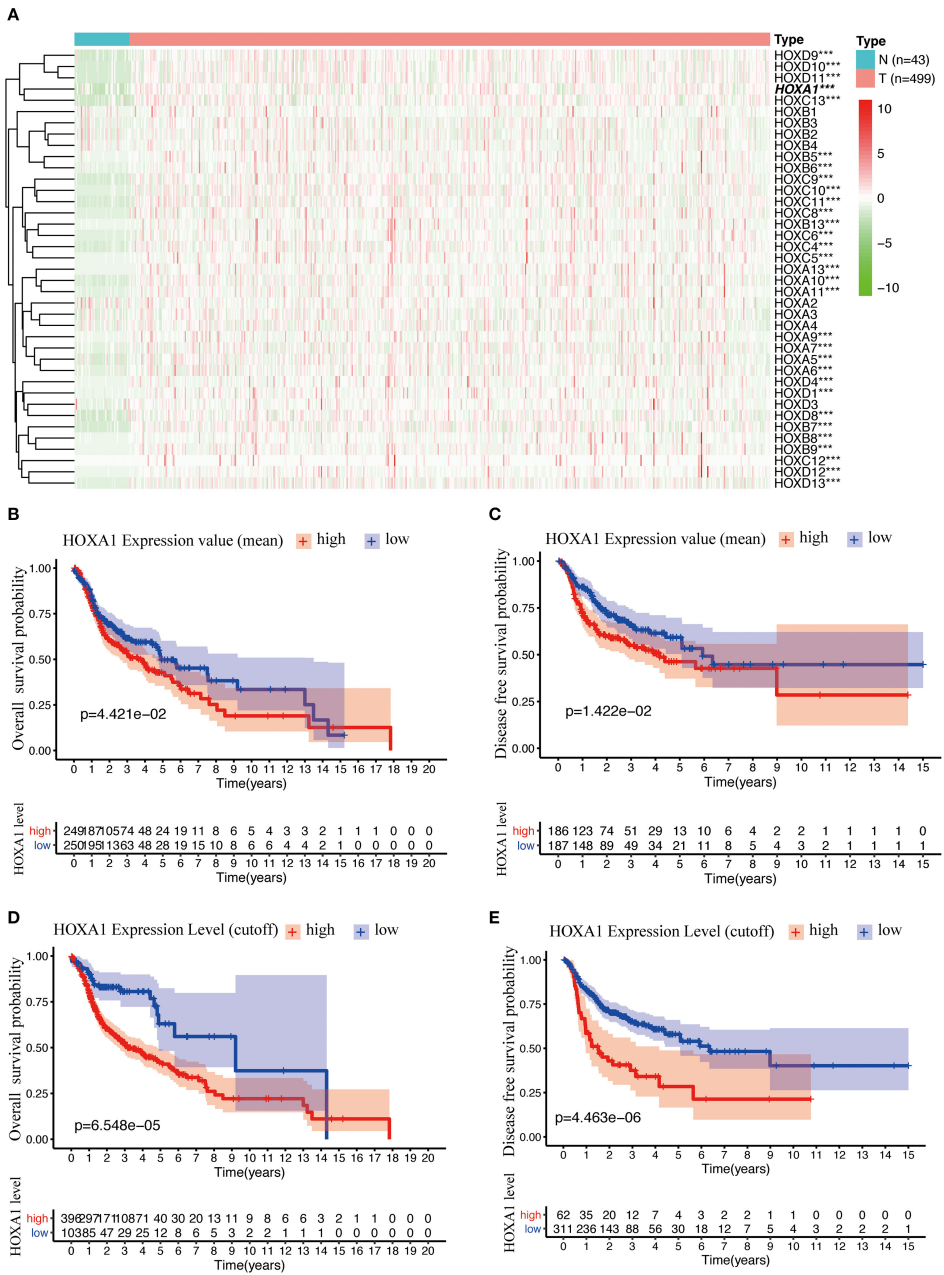
Differentially Expression and Prognostic Value OF HOX Genes in HNSCC. **(A)** Heatmap of HOX genes family included 30 differentially expressed genes (DEGs) in 499 tumor tissues compared with 43 para-carcinoma tissues based on TCGA-HNSC DATA. **(B)** Kaplan-Meier curve analysis of overall survival of TCGA-HNSC patients in high- and low-HOXA1 expression (mean value) groups (*p* = 4.421e−02). **(C)** Kaplan-Meier curve analysis of disease-free survival of TCGA-HNSC patients in high- and low-HOXA1 expression (mean value) groups (*p* = 1.422e−02). **(D)** Kaplan-Meier curve analysis of overall survival of TCGA-HNSC patients in high- and low-HOXA1 level (cut-off value) groups (*p* = 6.548e−05). **(E)** Kaplan-Meier curve analysis of disease-free survival of TCGA-HNSC patients in high- and low-HOXA1 level (cut-off value) groups (*p* = 4.463e−06). ***P < 0.001.

The analysis flow chart of this article is shown in Figure 1. Most Hox genes (30/39, Supplementary Material 3) have significant differences in expression between 499 tumor samples and 43 tumor-adjacent normal samples (Figure 2A). Among DEGs, only HOXA1 have predictive ability both in OS and DFS (Supplementary Figure 1), and worse clinical outcomes in patients with high expression of HOXA1 (Figures 2B–E).

### HOXA1 Is Upregulated in HNSCC

**Figure 3:**
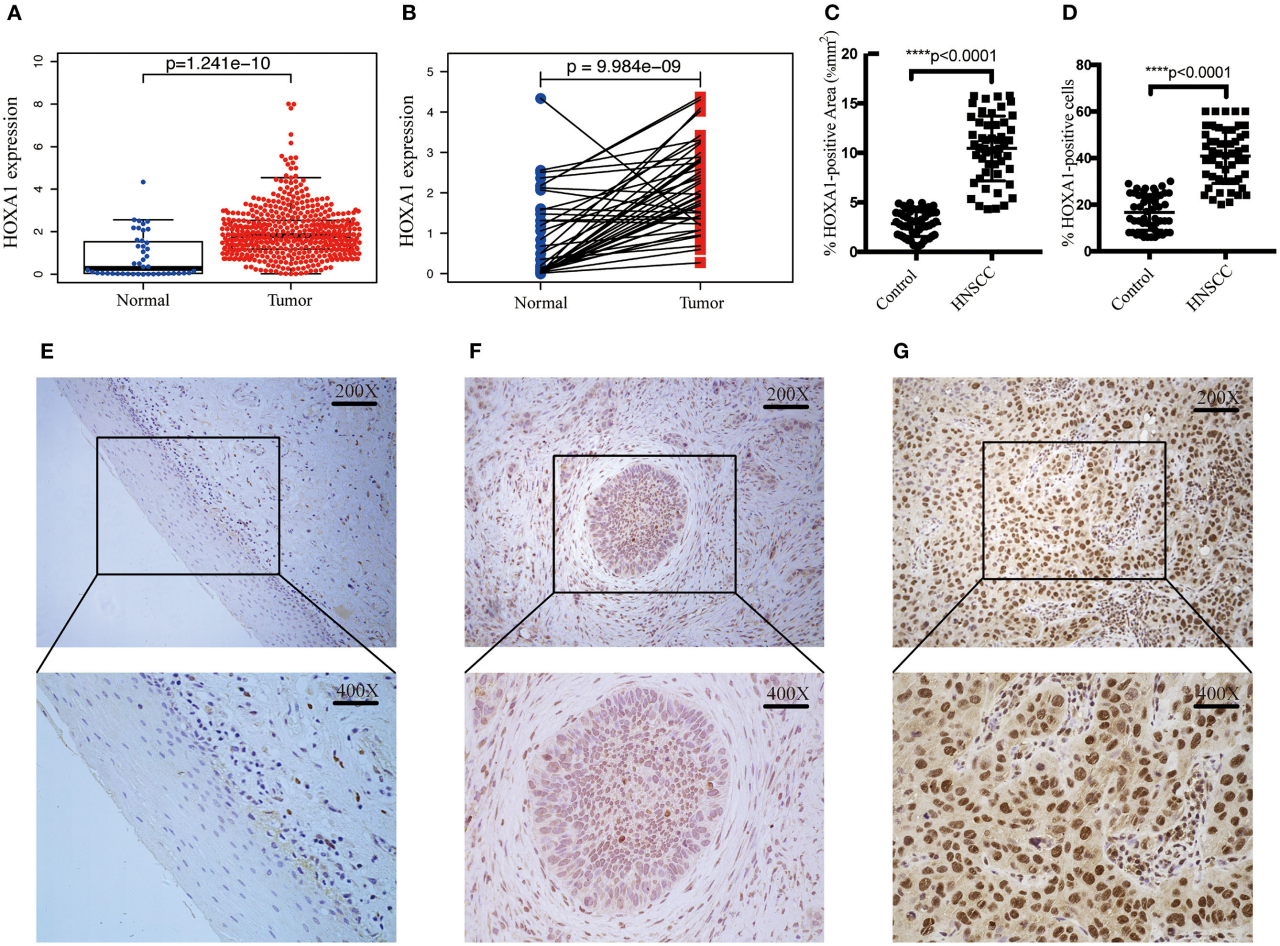
Transcriptional expression and protein level of HOXA1 is significantly high in HNSCC patients. **(A)** Higher HOXA1 mRNA expression in 499 tumor tissues compared with 43 para-carcinoma tissues based on TCGA-HNSC DATA (*p* = 1.241e−10). **(B)** Paired difference analysis of HOXA1 mRNA expression based on TCGA-HNSC DATA (*p* = 9.984e−09). **(C)** The percentage of HOXA1-positive area was significantly higher in HNSCC sections than in para-carcinoma sections (*P* < 0.0001). **(D)** The percentage of HOXA1-positive cells was significantly higher in HNSCC sections than in para-carcinoma sections (*P* < 0.0001). **(E–G)** Representative immunohistochemical staining for HOXA1 protein in 52 matched HNSCC tissues and para-carcinoma tissues. **(E)** Negative expression of HOXA1 in para-carcinoma tissues (−). **(F)** Low expression of HOXA1 protein in HNSCC specimens (− to +). **(G)** High expression of HOXA1 protein in primary HNSCC specimens (++). Original magnification: x200 (top), x400 (bottom).

In TCGA-HNSC, HOXA1 expression in tumor tissues was significantly higher than adjacent tissues (p = 1.241e-10) (Figure 3A), the differential analysis of HOXA1 expression in 43 paired samples got the same result (*p* = 9.984e−09) (Figure 3B). We performed IHC in 52 pairs of HNSCC paraffin sections for further verification (Figures 3E–G). HOXA1 protein immunostaining was mainly found in the cell nucleus but widely distributed in cancer nest. Among them, HOXA1 protein was detected in 45 cases (86.5%, + to ++) of tumor samples, while HOXA1 protein was detected in only ten sections (19.2%, + to ++) of adjacent pieces. There were 38 cases with low positive staining (− to +) and 14 patients with high positive staining (++) among the tumor samples. Our results also showed that HNSCC samples have a higher mean percentage of HOXA1-positive area (Figure 3C) and HOXA1-positive cells (Figure 3D) than paracancerous samples (*P* < 0.0001). The numeric dimension of the areas (mean ± SD, median) used for the IHC evaluation were shown in Table 2.

**Table 2:** The numeric dimension of the areas (mean ± SD, median) used for the IHC evaluation.

**The numeric dimension**	**Adjacent tissues** **(mean ± SD, median)**	**HNSCC tissues** **(mean ± SD, median)**	***P*-value**
HOXA1-positive area	2.86 ± 1.29, 2.94	10.44 ± 3.24, 10.48	<0.0001
HOXA1-positive cells	16.69 ± 7.60, 15.50	40.87 ± 11.62, 42.00	<0.0001

### Association of HOXA1 Expression With Clinicopathological Features

**Figure 4:**
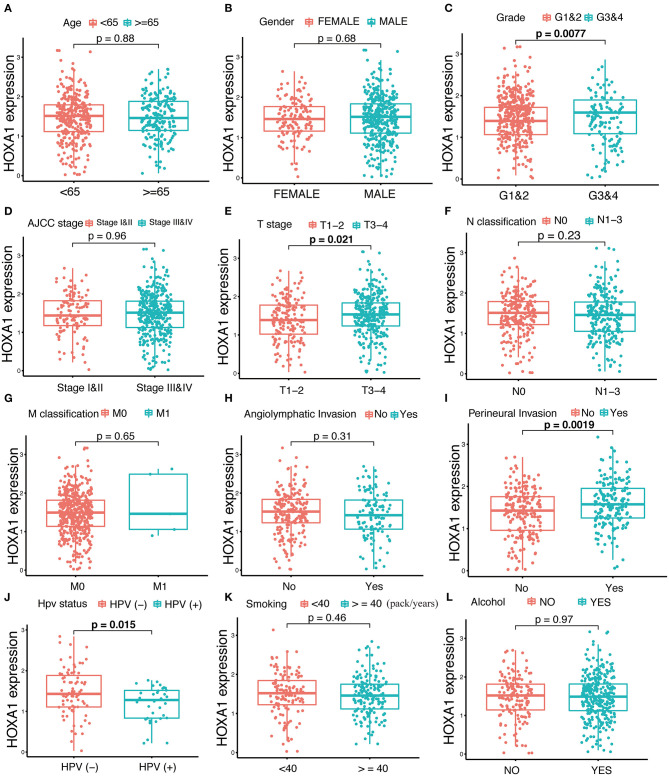
Correlation analysis between transcriptional expression of HOXA1 and clinicopathologic parameters in TCGA-HNSC patients. **(A–L)** HOXA1 was significantly correlated with Grade (G1and2 and G3and4, *p* = 0.0077), T stage (T1–2 and T3–4, *p* = 0.021), Perineural Invasion (No and Yes, *p* = 0.0019) and HPV Status (negative and positive, *p* = 0.015), while not significantly correlated with Age (<65 and ≥65 years, *p* = 0.88), *N* classification (N0 and N1-3, *p* = 0.23), M classification (M0 and M1, *p* = 0.65), Angiolymphatic Invasion (No and Yes, *p* = 0.31), Smoking (<40 and ≥40 packs/year, *p* = 0.46) or Alcohol (No and Yes, *p* = 0.97).

**Figure 5:**
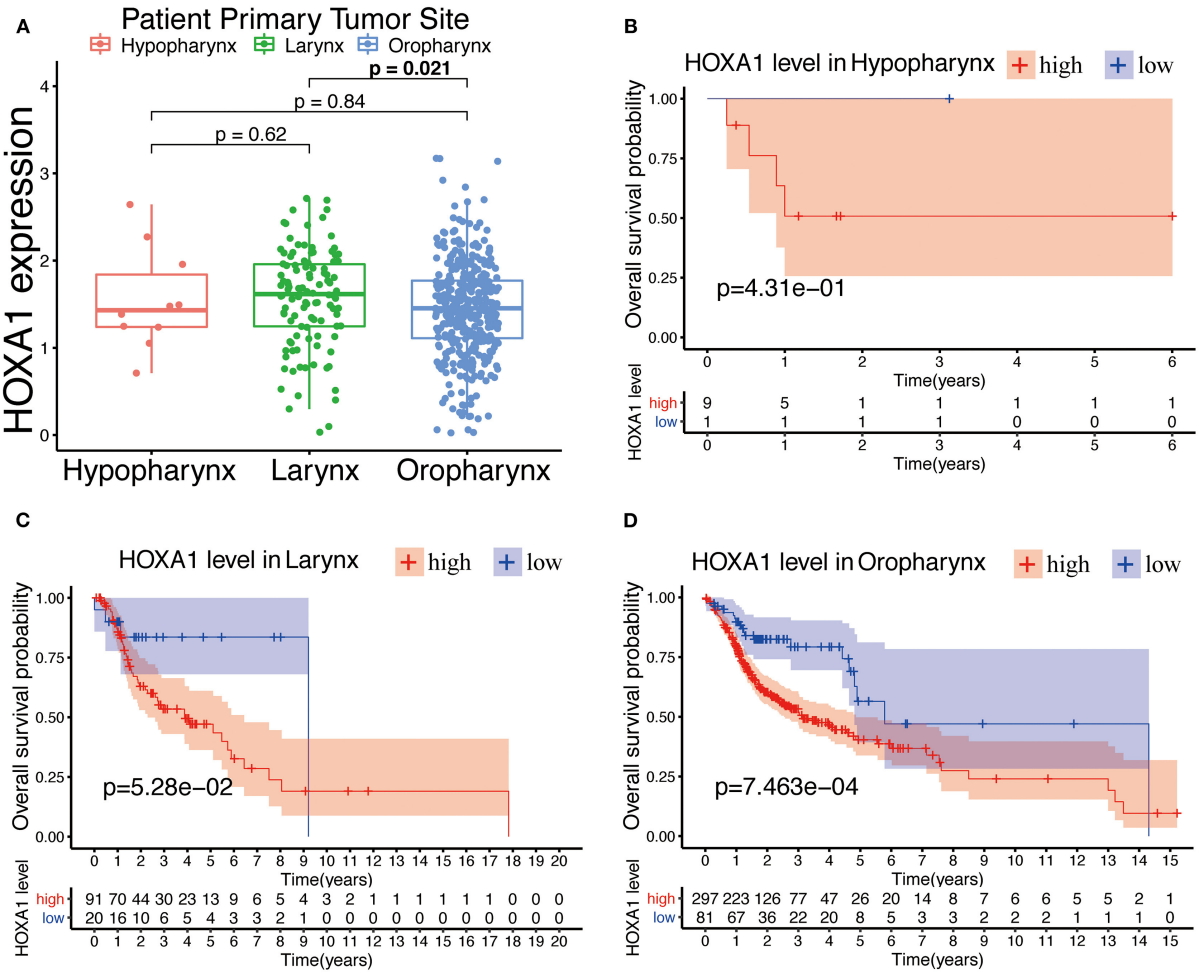
Prognostic performance of HOXA1 expression level for overall survival of different tumor site in HNSCC. **(A)** HOXA1 was significantly correlated with Primary Tumor Site (Larynx and Oropharynx, *p* = 0.021), while not significantly correlated with Primary Tumor Site (Hypopharynx and Larynx, *p* = 0.62 or Hypopharynx and Oropharynx, *p* = 0.84). Kaplan–Meier curve of the OS in the **(B)** Hypopharynx, **(C)** Larynx and **(D)** Oropharynx cohort stratified by the expression level of HOXA1.

The expression of HOXA1 was highly statistically significant correlated with pathology grade (G1&2 vs. G3&4, *p* = 0.0077), local invasion (T1–2 vs. 3–4, p = 0.023), perineural invasion (No vs. Yes, *p* = 0.0019) and HPV infection (positive vs. negative, *p* = 0.001) (Figures 4A–L). The expression of HOXA1 in oropharyngeal cancer was significantly lower than that in laryngeal cancer (*p* = 0.021), and there was no significant difference between the other two tumor locations (Figure 5A). We conducted further survival analysis in three tumor locations and found that the expression of HOXA1 only has prognostic value in oropharyngeal cancer (*P* < 0.05), while no significant predictive value in hypopharyngeal and laryngeal cancer (*p* > 0.05) in TCGA-HNSC patients (Figures 5B–D).

### Independent Predictive Power of HOXA1 Expression Value and Expression Level

**Figure 6:**
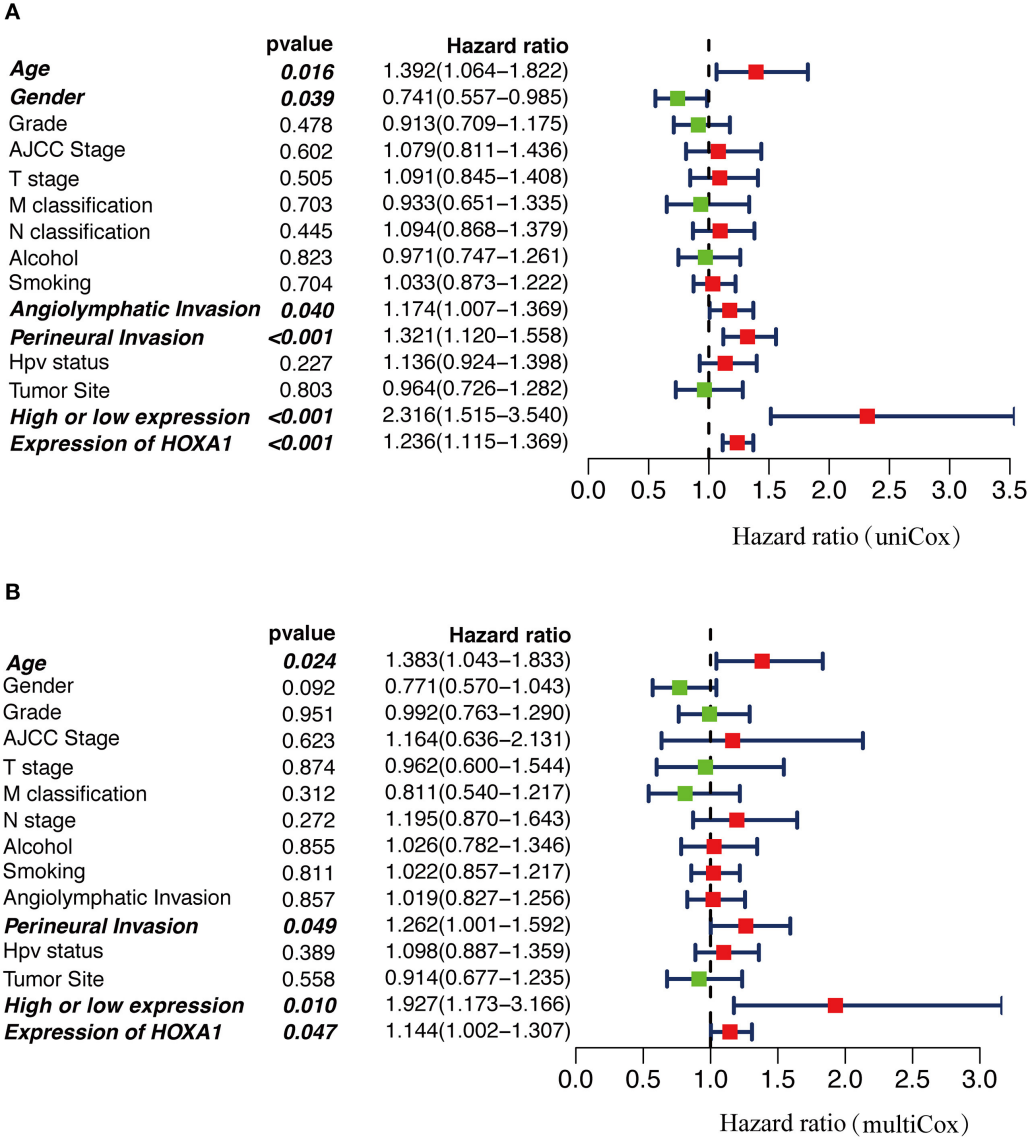
The Cox regression analysis for estimating the clinical value of HOXA1 expression value/expression level. **(A)** The univariate and **(B)** multivariate Cox regression analysis of age, gender, grade, AJCC stage, T stage, M classification, N classification, Smoking, Alcohol, Angiolymphatic Invasion, Perineural Invasion, HPV Status, Primary Tumor Site and HOXA1 expression value/expression level in TCGA-HNSC patients. Bold values indicate statistically significant, *P* < 0.05.

In Cox univariate regression analysis, the HRs of high-expression vs. low-expression of HOXA1 and expression value of HOXA1 for overall survival (OS) was 2.316 (*P* < 0.001, CI: 1.515–3.540) and 1.236 (*P* < 0.001, CI: 1.115–1.369), respectively (Figure 6A). In further multivariate Cox regression analysis, the expression level and expression value of HOXA1 still have a good predictive ability for OS [expression level (HR = 1.927, *p* = 0.010, CI: 1.173–3.166) and expression value (HR = 1.144, *p* = 0.047, CI: 1.002–1.307)] (Figure 6B). The results of the above analysis indicated HOXA1 was an independent prognostic factor, and the expression level of HOXA1 seem better.

### GSVA and GSEA Reveal HOXA1-Related Pathways and Molecular Functions

**Figure 7:**
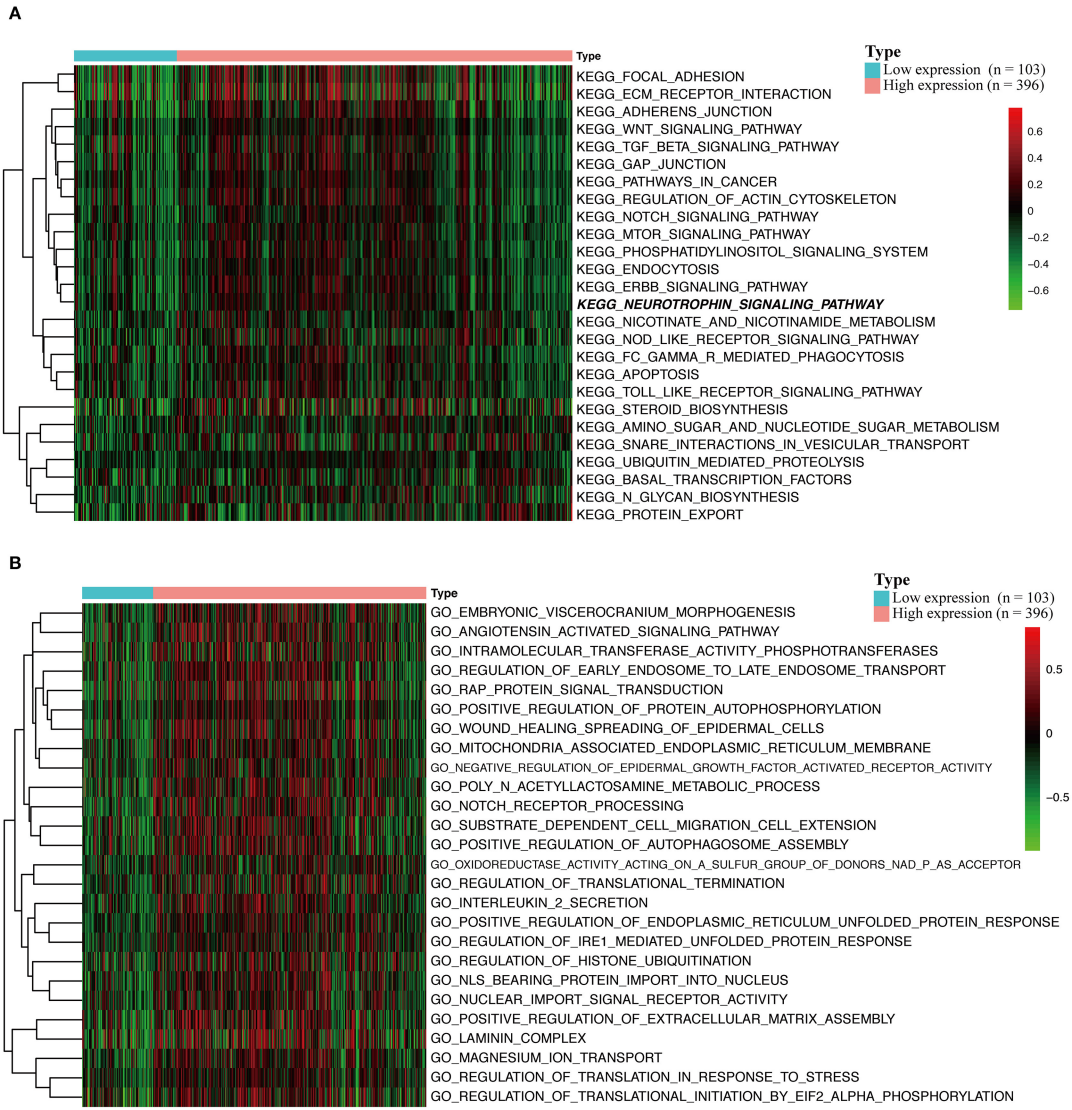
Gene set variation analysis (GSVA) of HOXA1 in TCGA-HNSC dataset. **(A)** GSVA-derived clustering heatmaps of differentially expressed pathways for high and low expression level of HOXA1. **(B)** GSVA-derived clustering heatmaps of differentially expressed Gene Ontology for high and low expression level of HOXA1.

**Figure 8:**
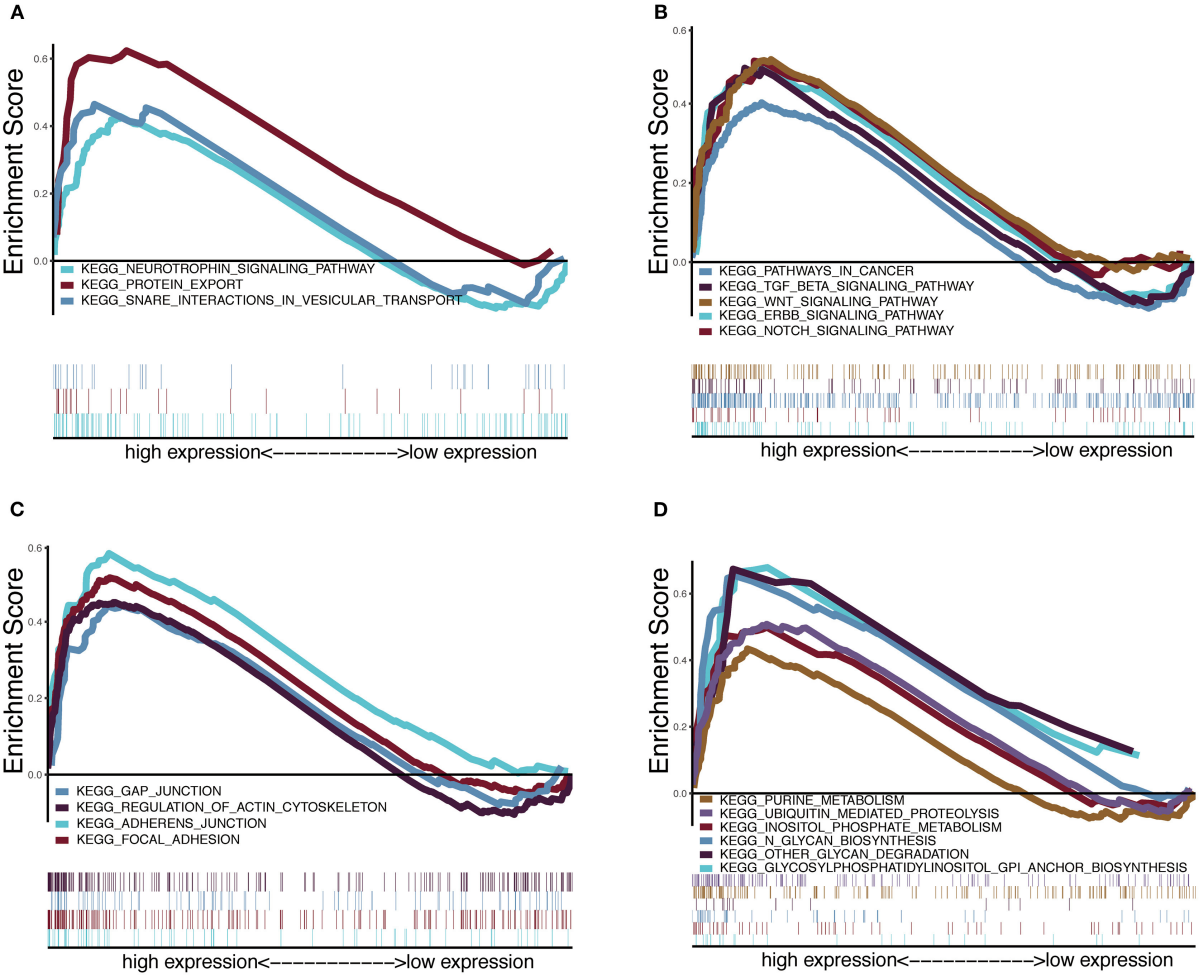
Enrichment plots from multiple-GSEA correlated with high expression value of HOXA1. **(A)** Neuroprotein secretion and transport: Neurotrophin signaling pathway, Protein export and Snare interactions in vesicular transport. **(B)** Tumor-associated Signaling Pathway: Pathways in cancer, Wnt signaling pathway, ERBB signaling pathway, Notch signaling pathway and TGF-BETA signaling pathway. **(C)** Cell adhesion and junction: Adheres junction, Regulation of actin cytoskeleton, Gap junction and Focal adhesion **(D)** Metabolic reprogramming: Glycosylphosphatidylinositol Gpi anchor biosynthesis, Ubiquitin proteolysis, Glycan biosynthesis, Purine metabolism, Glycan degradation and Inositol phosphate metabolism.

To explore the potential carcinogenic function of HOXA1, we used both GSVA and GSEA both approaches. After scoring 499 patients by the GSVA, the significant differences in signaling pathways and GO functions associated with the expression level of HOXA1 were obtained, as shown in Figures 7A,B. Besides, we ran GSEA to figure out the significant differential signaling pathways related to the expression value of HOXA1 (Table 3), including neuroprotein secretion and transport, the tumor-associated signaling pathway, cell adhere junction and metabolic reprogramming (Figures 8A–D). After the intersection of the results of GSVA and GSEA, 14 mutual pathways mainly focusing on the following four aspects: neurotrophin, vesicular transport, protein export; Ubiquitin mediated proteolysis, Glycan biosynthesis; gap junction, adherens junction, focal adhesion, regulation of actin cytoskeleton; Pathways in cancer including WNT/ERBB/ NOTCH/TGF-beta.

**Table 3:** Gene sets enriched related with expression of HOXA1.

**MSigDB collection**	**Gene set name**	**NES**	**NOM p-val**	**FDR q-val**
c2.cp.kegg.v7.1.symbols.gmt	KEGG_WNT_SIGNALING_PATHWAY	1.961	0	0.075
	KEGG_GLYCOSYLPHOSPHATIDYLINOSITOL_GPI_ANCHOR_BIOSYNTHESIS	1.952	0.002	0.054
	KEGG_ERBB_SIGNALING_PATHWAY	1.760	0.003	0.137
	KEGG_UBIQUITIN_MEDIATED_PROTEOLYSIS	1.829	0.004	0.100
	KEGG_N_GLYCAN_BIOSYNTHESIS	2.057	0.004	0.038
	KEGG_ADHERENS_JUNCTION	1.875	0.005	0.079
	KEGG_PURINE_METABOLISM	1.679	0.006	0.141
	KEGG_OTHER_GLYCAN_DEGRADATION	1.758	0.008	0.125
	KEGG_REGULATION_OF_ACTIN_CYTOSKELETON	1.712	0.015	0.135
	KEGG_PROTEIN_EXPORT	1.720	0.019	0.138
	KEGG_NOTCH_SIGNALING_PATHWAY	1.664	0.026	0.132
	KEGG_GAP_JUNCTION	1.614	0.028	0.155
	KEGG_TGF_BETA_SIGNALING_PATHWAY	1.674	0.029	0.137
	KEGG_FOCAL_ADHESION	1.696	0.031	0.132
	KEGG_INOSITOL_PHOSPHATE_METABOLISM	1.629	0.034	0.161
	KEGG_PATHWAYS_IN_CANCER	1.591	0.038	0.146
	KEGG_NEUROTROPHIN_SIGNALING_PATHWAY	1.623	0.039	0.160
	KEGG_SNARE_INTERACTIONS_IN_VESICULAR_TRANSPORT	1.588	0.045	0.142

*Gene sets with NOM P-value < 0.05 and FDR q-value < 0.25 are considered as significant*.

*MsigDB, Molecular Signatures Database; NES, normalized enrichment score; NOM, nominal; FDR, false discovery rate*.

### Immune Infiltration and DNA Methylation Correlating With HOXA1 Expression

**Figure 9:**
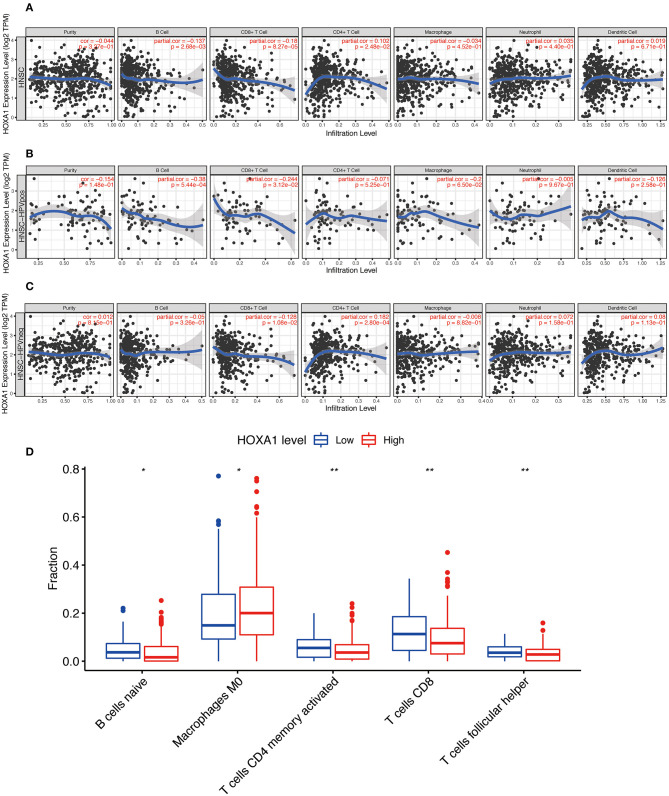
Immune Infiltration Correlating with HOXA1 Expression in TCGA-HNSC dataset. Association of HOXA1 expression with immune infiltration in **(A)** all HNSCC patients, **(B)** HNSCC-HPV_POS_ patients and **(C)** HNSCC-HPV_neg_ patients based on TIMER database. **(D)** The association of statistically significant immune cells infiltration and the HOXA1 expression in CIBERSORT analysis. Note: *P < 0.05, **P < 0.01.

More and more studies have found that some oncogenes significantly affect the tumor immune microenvironment, including infiltration of B cell, CD8+ T cell, CD4+ T cell, Macrophage, Neutrophil, Dendritic cell, etc. The HOXA1 expression in the TIMER database is associated with a decrease of B cell / CD8+T cell infiltration and an increase of CD4+ T cell infiltration in total HNSCC patients, a reduction of B cell and CD8+T cell infiltration in HPV(+) HNSCC patients, and a decrease of CD8+T cell infiltration and an increase of CD4+ T cell infiltration in HPV(−) HNSCC patients (Figures 9A–C). From the results of immune infiltration in total and HPV subtype HNSCC patients, it is found that the high expression of HOXA1 significantly leads to the reduction of CD8+ T cell infiltration. Besides, we performed the CIBERSORT analysis to screen the differential TICs of the high and low-HOXA1 expression groups and found that the results were similar to the TIMER analysis results. The CIBERSORT analysis results showed that the proportion of M0 macrophages was positively correlated with HOXA1 expression level, and the ratio of CD8+T cells, naïve B cells, CD4 memory activated T cells and follicular helper T cells were negatively associated with the level of HOXA1 expression (Figure 9D).

**Figure 10:**
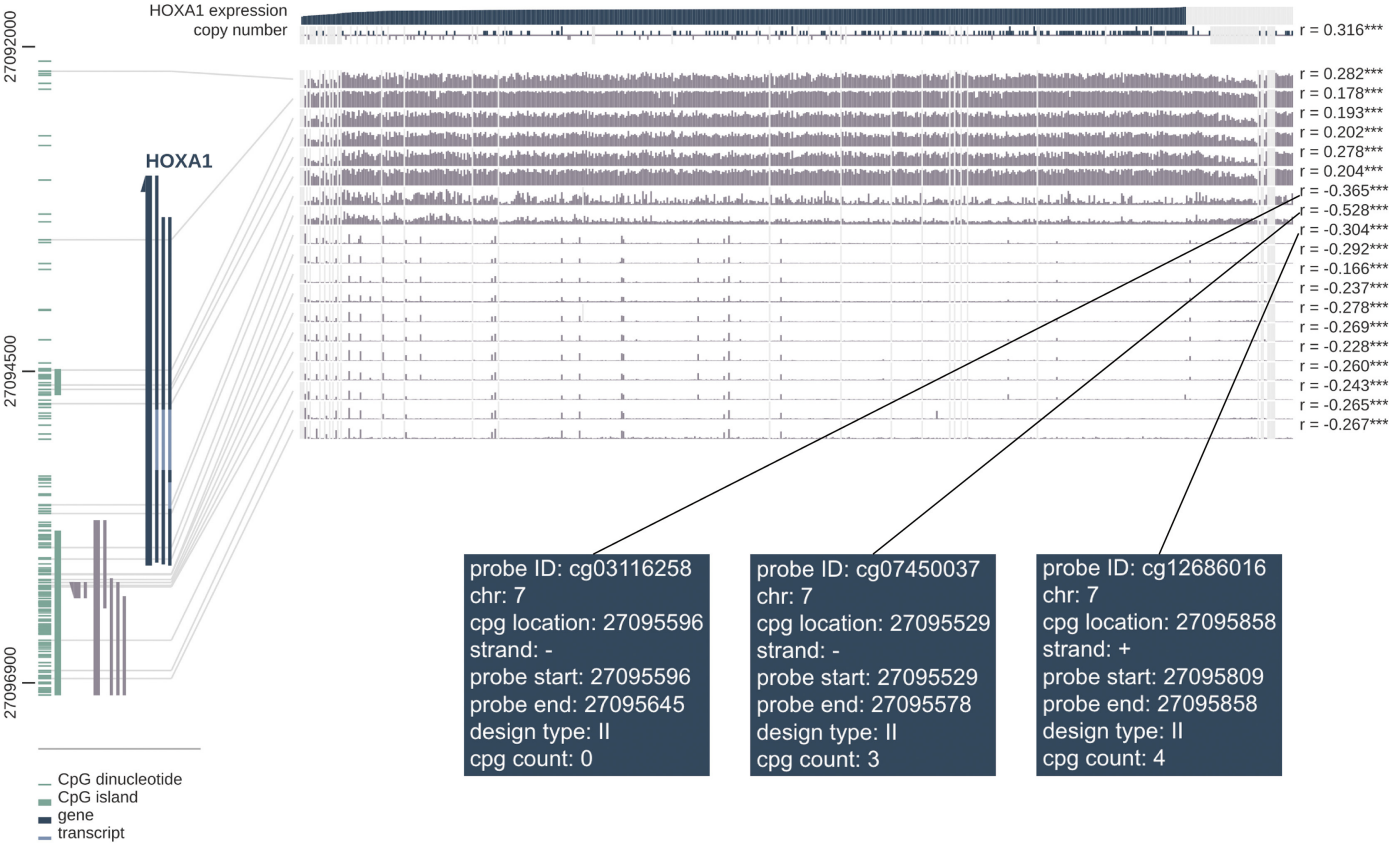
MEXPRESS visualization of the TCGA data for HOXA1 in HNSCC. This figure shows the complex interplay between gene expression, copy number, DNA methylation and probes ID for HOXA1 in HNSCC. Pearson's correlation coefficients and *P*-values from Wilcoxon rank-sum test for methylation sites and HOXA1 expression are shown on the right side. The gray lines stand for Infinium 450 k probes and their heights represent the beta value for this probe. Dark gray and green lines at the bottom left indicate the HOXA1 and CpG islands.

The correlation analysis between DNA methylation level and HOXA1 expression showed that high HOXA1 expression with less promoter methylation in tumor samples (Pearson correlation coefficients ranging from −0.166 to −0.528 for promoter region probes) in the MEXPRESS database (Figure 10). We also found that the probes such as cg03116258, cg07450037 and cd12686016 of the seventh chromosome had the strongest correlation in DNA methylation of HOXA1, which was worthy of further exploration and research.

## Discussion

There are only sporadic reports in the literature on the role of HOX genes in HNSCC. Jinyun Li et al. used bioinformatics to analyze the HOXA genes' role in laryngeal squamous cell carcinoma (LSCC), in which HOXA10, HOXA11 and HOXA13 expression and clinical characteristics (M stage and gender) are independent prognostic biomarkers for the prognosis of LSCC patients (Li et al., 2020). The expression of HOXA members in LSCC is negatively correlated with the level of methylation. HOXA7, HOXA10 and HOXA13, which are highly expressed in LSCC tissues, have a weak positive correlation with tumor purity. However, there is no significant correlation between the TICs and HOXA members. Studies in oral squamous cell carcinoma (OSCC) showed that HOXA genes are more enriched than normal mucosal tissues. High expression of HOXA1 can promote OSCC proliferation and maybe an independent prognostic indicator for patients (Bitu et al., 2012). Both AMOT-p80 and miR-146a, identified as HOXD10 targets in HNSCC, can represent therapeutic targets for specific tumor stages (Hakami et al., 2014). High expression of HOXD10 promotes tumor proliferation and migration, while low expression of HOXD10 can promote tumor invasion and metastasis by regulating AMOT-p80. In this paper, we showed the expression profile of 39 members of the HOX genes in HNSCC, as well as the most important independent prognostic indicator and potential biological role of HOXA1 in HNSCC, providing novel insights for further research on HOXA1 as a potential target of HNSCC.

The expression of HOXA1 is tissue-specific (Fagerberg et al., 2014) (https://www.proteinatlas.org), and as tumor modulators, it may cover a wide range of functionality in types of tumor dependent on the cellular context (Shah and Sukumar, 2010). HOXA1 played a role of an oncogene in breast carcinoma (Liu et al., 2019), lung carcinoma (Abe et al., 2006), oral squamous cell carcinoma (Bitu et al., 2012), hepatocellular carcinoma (Zha et al., 2012), melanoma (Wardwell-Ozgo et al., 2014), gastric cancer (Yuan et al., 2016), leukemia (Chen et al., 2019) and retinoblastoma (RB) (Lyv et al., 2020). Inversely, it is shown as anti-oncogenes in pancreatic carcinoma (Ohuchida et al., 2012) and small cell lung cancer (SCLC) (Xiao et al., 2014). Nilay et al. pointed out that the abnormal expression of Hox genes triggers oncogenesis or tumor suppression, which may be caused by her mechanism of action (Shah and Sukumar, 2010). On the level of expression, Hox genes are associated with oncogenesis when expressed at an increased level in tumor tissues not normally seen in that tissue type. In the results of our HNSCC IHC, the expression of HOXA1, which is mainly located in the cell nucleus, is extremely low in adjacent normal tissues while it is widely distributed in cancer nest. Thus, it can be seen that the abnormal up-regulation of HOXA1 in HNSCC, especially in the nucleus, indicates that HOXA1 mainly plays a role in oncogenesis.

E-cadherin activated Rac signaling pathway through the transcription target of HOXA1 (Zhang et al., 2006), which can promote breast cancer cell survival by upregulating cyclinD1, c-Myc, and BCL-2 (Mohankumar et al., 2007, 2008). HOXA1 also takes effect independent of transcriptional regulation (Taminiau et al., 2016). In proteomics research, it is found that HOXA1 also participates in cell signal transduction as a functional protein, such as pathways of focal adhesion, receptor tyrosine kinase function, TGF/BMP and TNF/NF-kB (Lambert et al., 2012). In breast cancer, HOXA1 transforms immortalized human breast epithelial cells stimulated by p44/42 MAPK pathway, signal transducer and activator of transcription 3 (STAT3) and STAT5B into an invasive malignant phenotype. WNT, TGF-beta and FGF pathways may synergistically affect HOXA1 expression, and WNT7A directly maintains expression of HOXA1, making for lung cancer recurrence (Calvo et al., 2000). HOXA1 dedifferentiates melanoma cells into a highly aggressive cell state accompanied by TGF activation (Wardwell-Ozgo et al., 2014). Down-regulation of HOXA1 can increase the expression of E-cadherin while reducing the expression of Snail and MMP-3, which can inhibit ERK1/2 and AKT to suppress the growth, migration, invasion and metastasis of prostate cancer (Wang et al., 2015).

The methylation of DNA is inversely proportional to transcription activity, which also affects the genome's stability. Yeon et al. evaluated the methylation status of 15 genes of promoter CpG island, including HOXA1, that are involved in breast cancer progression (Park et al., 2012). The expression level of HOXA1 in subtypes of breast cancers are related to the disordered epigenetic activity, in which the methylation frequency of Luminal B is the highest (< 0.001). Meanwhile, HOXA1 hypermethylation is related to the absence of HER2 neu expression and proliferation of breast cancer cells (*P* < 0.05) (Pilato et al., 2013). Through the MEXPRESS database, we found that more HOXA1 expression in HNSCC samples with less promoter methylation, which possibly foreshadowing the lower DNA methylation level of oncogenes, may lead to the occurrence of tumors.

In addition to DNA methylation, many non-coding RNAs (ncRNAs) as another way of apparent epigenesis have been found to participate in the control of gene expression and activation of pathways, which in turn participates in the development of cancer and other diseases. Many studies have shown that circRNAs, lncRNAs and miRNAs play a key role in tumorigenesis, invasion, metastasis and drug resistance through the function of competitive RNA (ceRNA). Here we summarize Non-coding genes act as regulators of HOXA1 in cancers (Table 4).

**Table 4:** Non-coding genes act as regulators of HOXA1 in cancers.

**Non-coding form**	**Non-coding RNAs**	**Cancer types**	**Functions**	**Ref. (PMID)**
microRNA	miR-193a-5p	Breast cancer	Tumor promotor	32497022
	miR-100	Lung cancer	Chemotherapy resistance	24559685
		Nasopharyngeal Carcinoma	Growth and proliferation	32021301
	miR-218	Lung cancer	Gefitinib Resistance	32084702
		Cervical cancer	Proliferation, colony formation, migration and invasion	30896864
	miR-181b	SCLC	Regulatory of p53 signaling pathway	29658571
	miR-181a-5p	MM	Apoptosis resistance	29228867
	miR-216b-5p	Cervical cancer	Proliferation	31114990
	miR-210	SCLC/melanoma	Immune escape	22962263
	miR-145	Oral squamous cell carcinoma	Cancer inhibition	31138758
	miR-10a	PC	Tumor promotor	22407312
	miR-30c	Malignant giant-cell tumor of bone	Metastasis and growth	29164581
	miR-30b	Esophageal cancer	Growth, migration and invasion	28189678
	miR-99a	HCC	Migration and invasion	31186723
	miR-99 family	Epithelial cell	Proliferation and migration	24312487
	miR-515	RB	Tumor promotor	32561925
LncRNA	LncRNA SNHG1	Breast cancer	Tumor promotor	32497022
	LncRNA CCAT1	Lung cancer	Gefitinib Resistance	32084702
		MM	Apoptosis resistance	29228867
	LINC00152	Cervical cancer	Proliferation	31114990
	lncRNA HOTAIR	SCLC	Multidrug resistance	26707824
	LncRNA HOTAIRM1	Breast cancer	Avoiding silence of HOXA1	32284737
		Endometrial Cancer	Proliferation, Migration, Invasion and EMT	31853186
		GBM	Growth and invasion	30376874
	LncRNA ZFPM2-AS1	RB	Tumor promotor	32561925
CircRNA	CircEIF4G2	Cervical cancer	Proliferation, colony formation, migration and invasion	30896864

*SCLC, small cell lung cancer; RB, retinoblastoma; GBM, glioblastoma multiforme; PC, Pancreatic cancer; MM, Multiple Myeloma; HCC, hepatocellular carcinoma cells; EMT, epithelial–mesenchymal transition*.

Tumor immunity research is in full swing, and we are still full of unknowns on how the abnormal expression of HOXA1 affects immune function. In the study of endometrial cancer, high expression of HOXA1 in Myeloid-derived suppressor cells (MDSCs) promotes tumor progression and hinders anti-tumor immune response (Li et al., 2019). Tumors adapt to hypoxic stress and create an immunosuppressive microenvironment suitable for survival (Keith et al., 2011). Studies have found that in hypoxic areas of SCLC and melanoma, miR-210 regulates the expression of HOXA1 and inhibits the anti-tumor effect of cytotoxic T lymphocyte (Noman et al., 2012). Regarding the results of TIMER and CIBERSORT analysis, we suspected that the high expression of HOXA1 in HNSCC might reduce CD8+ T cell infiltration, leading to an immunosuppressive environment not conducive to patient's survival outcomes.

Previous studies of pancreatic cancer and glioma showed that the close interaction between tumors and neurons is not conducive to the clinical prognosis. The density of newborn nerve cells in the tumor microenvironment is often associated with tumor growth and highly invasive biological behaviors (Magnon et al., 2013; Venkataramani et al., 2019). The latest study by Moran Amit et al. found that the loss of TP53 in HNSCC can promote tumor growth by affecting neuronal remodeling (Amit et al., 2020). So far, we have found that the function of p53 in SCLC and osteosarcoma is related to the regulation of HOXA1 (Zhao et al., 2015; Zhang et al., 2018). In mouse development studies, it was found that at embryonic stage (D7-9), HOXA1 expression precisely regulates the development of neural crest precursor cells, which are essential for tumor microenvironment remodeling (Gavalas et al., 2001). In this paper, we found that high HOXA1 expression is related to PNI by mining TCGA-HNSC data. According to the analysis of GSVA and GESA, we speculated that HNSCC might act on neurons by secreting exosomes containing neurotrophic proteins. However, this result lacked evidence concerning that no cases that meet PNI were found after careful screening of the paraffin blocks by pathologists. Whether HNSCC hijacks surrounding neurons to promote tumor growth through the regulation of HOXs is still worth exploring.

In summary, HOXA1 may mainly exert tumor regulation functions through the following four mechanisms: spatiotemporal expression disorder (high expression in cancer tissues, especially in the cell nucleus), abnormal protein-signaling pathways (neuronal protein secretion), mediation of immunosuppression (inhibition of CD8+ T cell infiltration) and aberrant epigenetic activity (high expression of HOXA1 leads to a decrease in DNA methylation level). Our study demonstrated that HOXA1 is highly expressed in HNSCC, which may be an independent prognostic factor in TCGA-HNSC patients, especially for oropharyngeal cancer patients. At present, HOXA1 small interfering RNA (siRNA) nanoparticles have achieved initial success in mouse models of breast cancer and are effective (75%) to reduce the incidence of tumors (Brock et al., 2014). More random cohort studies and mechanism experiments will help confirm the regulatory effects of HOXA1 on HNSCC.

## Data Availability Statement

Publicly available datasets were analyzed in this study. This data can be found here: https://portal.gdc.cancer.gov/repository.

## Ethics Statement

The studies involving human participants were reviewed and approved by Clinical Research Ethics Committee of The First Affiliated Hospital of Bengbu Medical College. The patients/participants provided their written informed consent to participate in this study.

## Author Contributions

The project was proposed by HL and SM. HL provided paraffin tissue sections and wrote the manuscript. MW assisted in editing the manuscript. JZ downloaded and mined the relevant data of TCGA. XW and MZ did immunohistochemistry and assisted in organizing data. All authors contributed to the article and approved the submitted version.

## Conflict of Interest

The authors declare that the research was conducted in the absence of any commercial or financial relationships that could be construed as a potential conflict of interest.

## Supplementary Material

Supplementary Figure 1 Kaplan-Meier survival analysis of **(A)** overall survival and **(B)** disease free survival) according to the expression of all HOX genes in TCGA-HNSC patients. The log-rank test was used to calculate the *p*-value.

Supplementary Material 1 CIBERSORT results of the fraction of twenty-two immune cell types of each TCGA-HNSC sample.

Supplementary Material 2 Clinicopathological characteristics of 52 HNSCC patients collected from The First Affiliated Hospital of Bengbu Medical College.

Supplementary Material 3 Results of differential expression analysis in all HOX genes.
