# Supplementary material for: Identification of HOXA1 as a Novel Biomarker in Prognosis of Head and Neck Squamous Cell Carcinoma
Source: Front Mol Biosci. 2021 Mar 8;7:602068. doi: 10.3389/fmolb.2020.602068 (PMC7982851; doi:10.3389/fmolb.2020.602068)
Supplement: Supplementary Figure 1 — Kaplan-Meier survival analysis of (A) overall survival and (B) disease free survival) according to the expression of all HOX genes in TCGA-HNSC patients. The log-rank test was used to calculate the p-value. [file Data_Sheet_1.PDF]

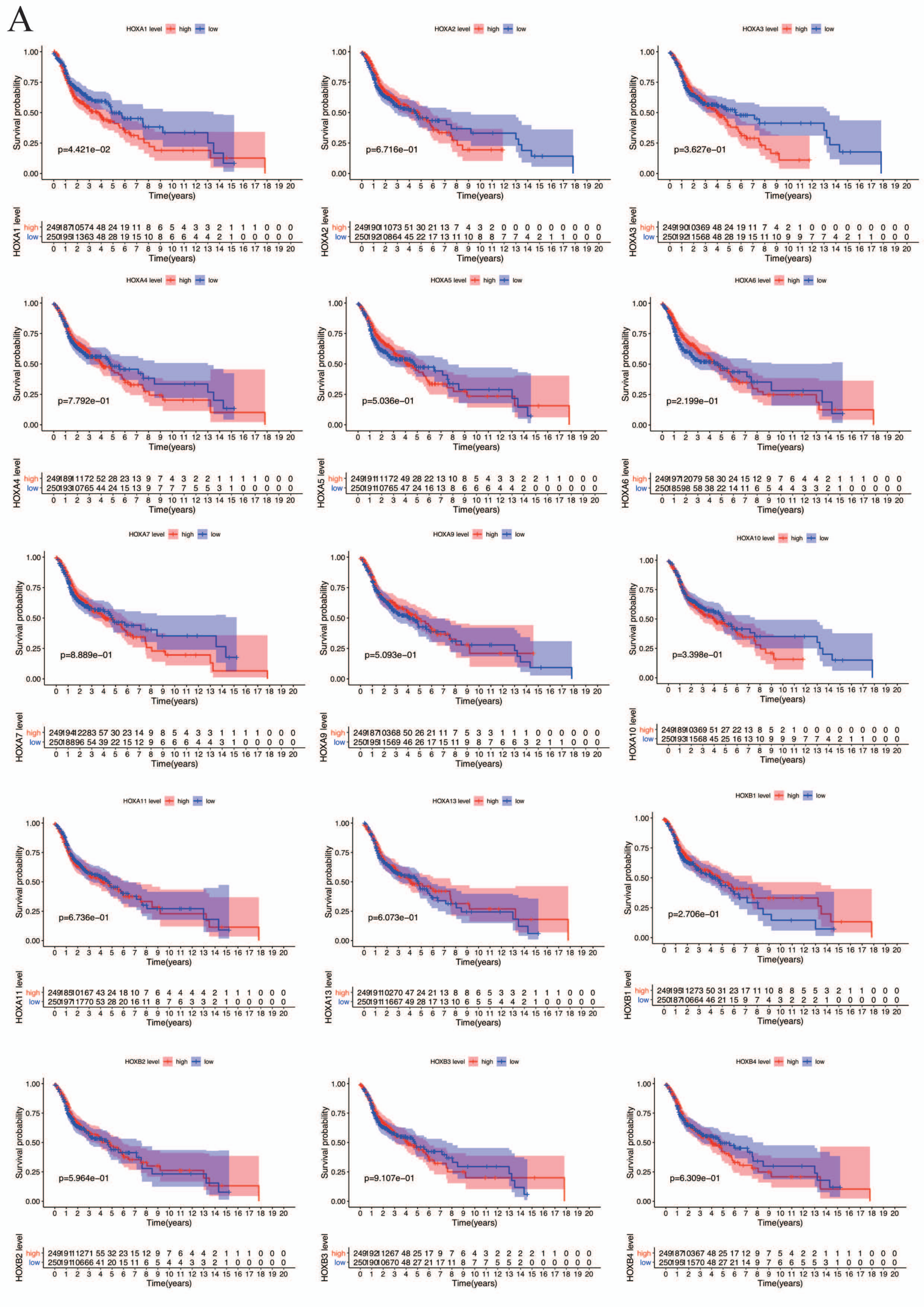
A
HOXA1 level high low
p=4.421e−02
HOXA2 level high low
p=6.716e−01
HOXA3 level high low
p=3.627e−01
HOXA4 level high low
p=7.792e−01
HOXA5 level high low
p=5.036e−01
HOXA6 level high low
p=2.199e−01
HOXA7 level high low
p=8.889e−01
HOXA9 level high low
p=5.093e−01
HOXA10 level high low
p=3.398e−01
HOXA11 level high low
p=6.736e−01
HOXA13 level high low
p=6.073e−01
HOXB1 level high low
p=2.706e−01
HOXB2 level high low
p=5.964e−01
HOXB3 level high low
p=9.107e−01
HOXB4 level high low
p=6.309e−01
Survival probability
Time(years)

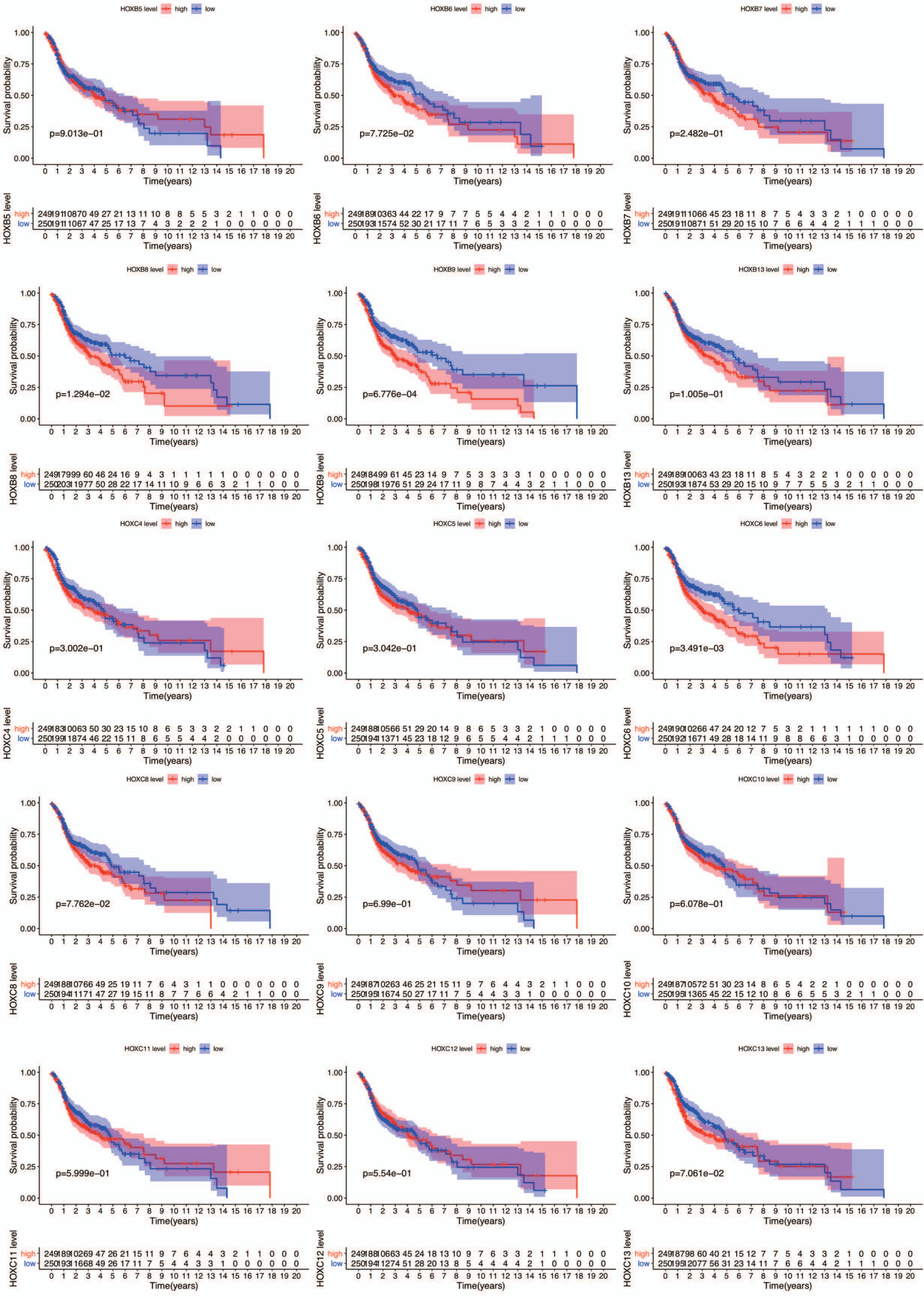
HOXB5 level high low
p=9.013e-01
HOXB6 level high low
p=7.725e-02
HOXB7 level high low
p=2.482e-01
HOXB8 level high low
p=1.294e-02
HOXB9 level high low
p=6.776e-04
HOXB13 level high low
p=1.005e-01
HOXC4 level high low
p=3.002e-01
HOXC5 level high low
p=3.042e-01
HOXC6 level high low
p=3.491e-03
HOXC8 level high low
p=7.762e-02
HOXC9 level high low
p=6.99e-01
HOXC10 level high low
p=6.078e-01
HOXC11 level high low
p=5.999e-01
HOXC12 level high low
p=5.54e-01
HOXC13 level high low
p=7.061e-02
Survival probability
Time(years)

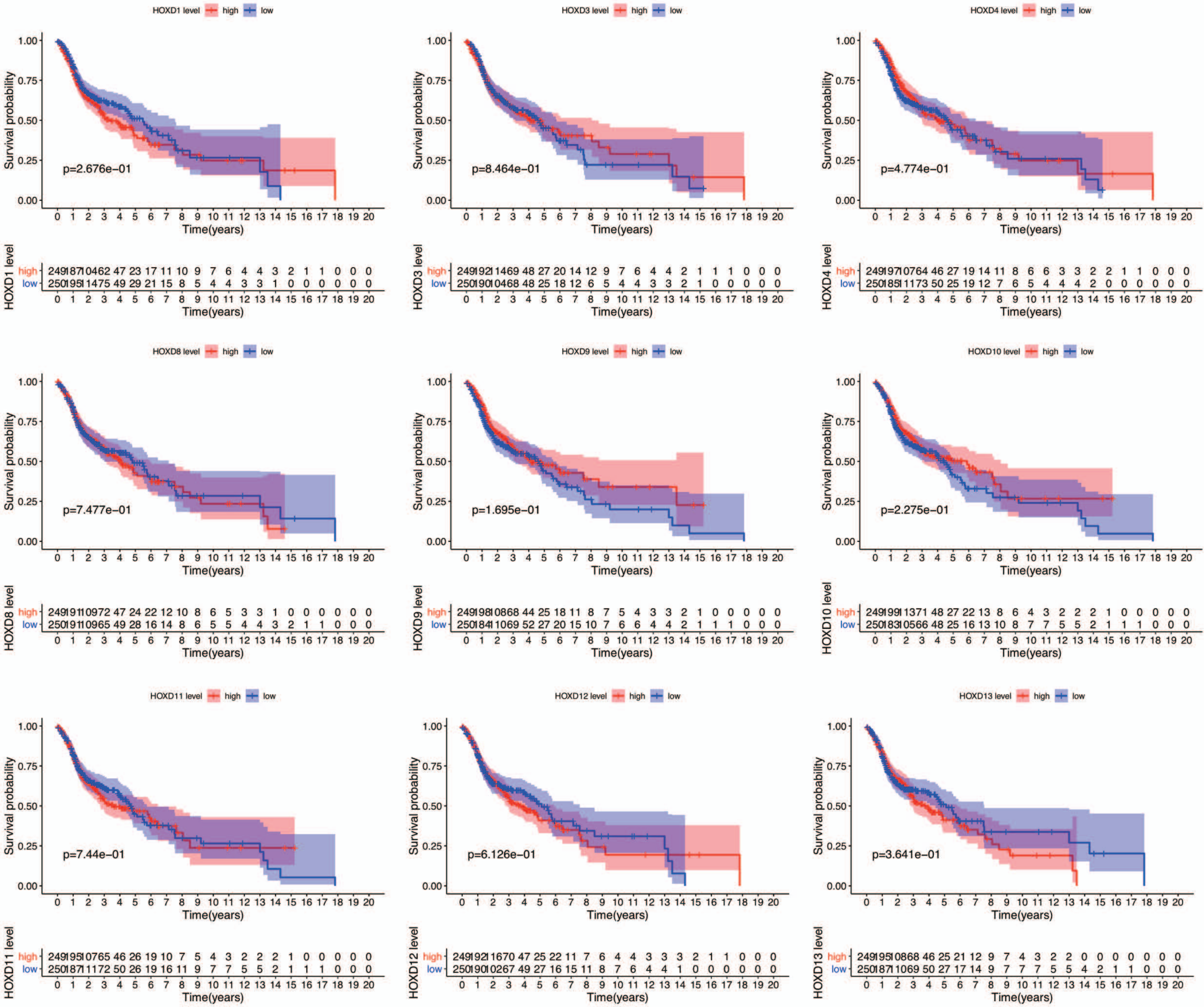
HOXD1 level high low
p=2.676e−01
HOXD3 level high low
p=8.464e−01
HOXD4 level high low
p=4.774e−01
HOXD8 level high low
p=7.477e−01
HOXD9 level high low
p=1.695e−01
HOXD10 level high low
p=2.275e−01
HOXD11 level high low
p=7.44e−01
HOXD12 level high low
p=6.126e−01
HOXD13 level high low
p=3.641e−01
Survival probability
Time(years)

B

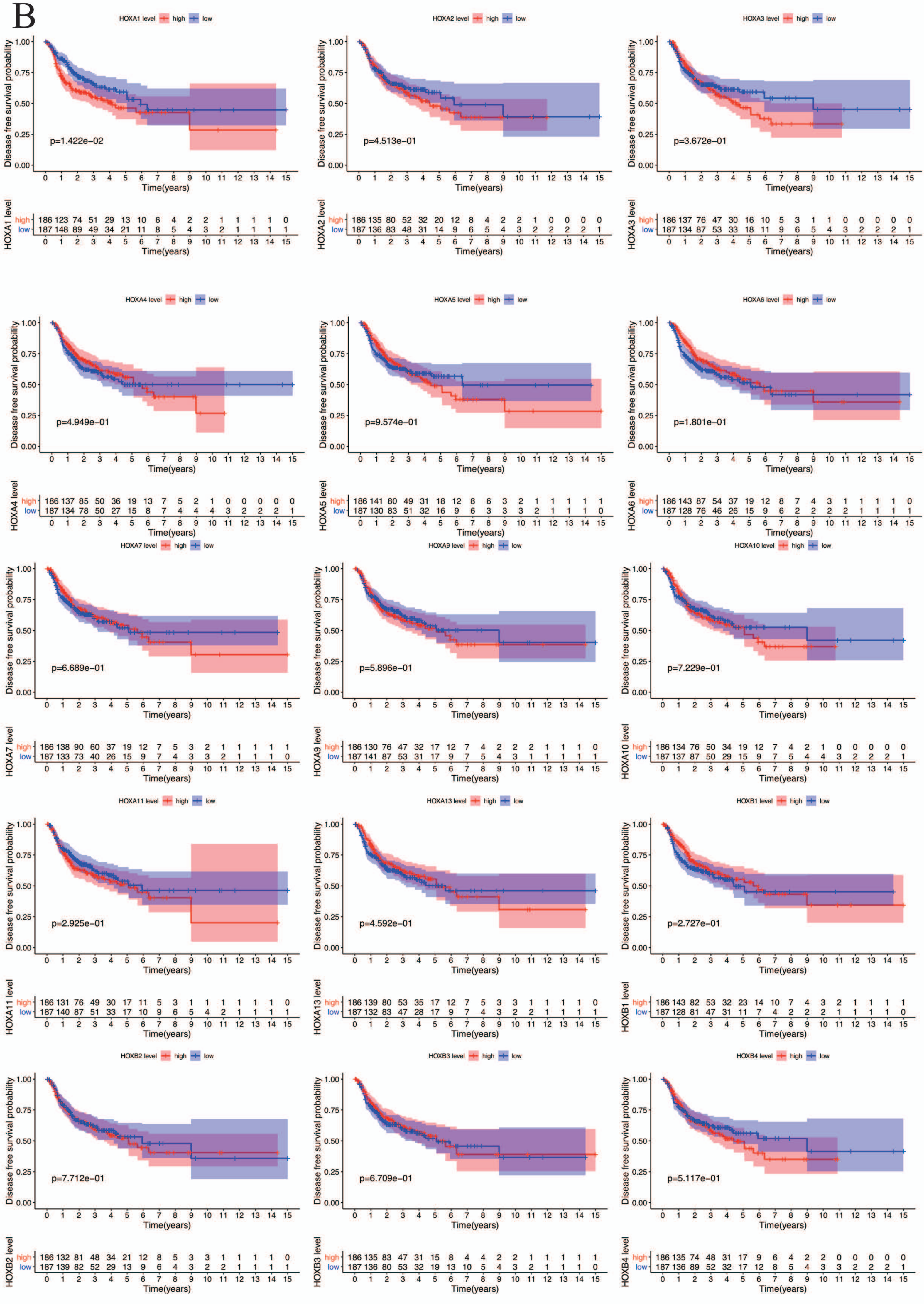

HOXA1 level high low
p=1.422e-02
high 186 123 74 51 29 13 10 6 4 2 2 1 1 1 1 0
low 187 148 89 49 34 21 11 8 5 4 3 2 1 1 1 1
HOXA2 level high low
p=4.513e-01
high 186 135 80 52 32 20 12 8 4 2 2 1 0 0 0 0
low 187 136 83 48 31 14 9 6 5 4 3 2 2 2 2 1
HOXA3 level high low
p=3.672e-01
high 186 137 76 47 30 16 10 5 3 1 1 0 0 0 0 0
low 187 134 87 53 33 18 11 9 6 5 4 3 2 2 2 1
HOXA4 level high low
p=4.949e-01
high 186 137 85 50 36 19 13 7 5 2 1 0 0 0 0 0
low 187 134 78 50 27 15 8 7 4 4 4 3 2 2 2 1
HOXA5 level high low
p=9.574e-01
high 186 141 80 49 31 18 12 8 6 3 2 1 1 1 1 1
low 187 130 83 51 32 16 9 6 3 3 3 2 1 1 1 0
HOXA6 level high low
p=1.801e-01
high 186 143 87 54 37 19 12 8 7 4 3 1 1 1 1 0
low 187 128 76 46 26 15 9 6 2 2 2 2 1 1 1 1
HOXA7 level high low
p=6.689e-01
high 186 138 90 60 37 19 12 7 5 3 2 1 1 1 1 1
low 187 133 73 40 26 15 9 7 4 3 3 2 1 1 1 0
HOXA9 level high low
p=5.896e-01
high 186 130 76 47 32 17 12 7 4 2 2 2 1 1 1 0
low 187 141 87 53 31 17 9 7 5 4 3 1 1 1 1 1
HOXA10 level high low
p=7.229e-01
high 186 134 76 50 34 19 12 7 4 2 1 0 0 0 0 0
low 187 137 87 50 29 15 9 7 5 4 4 3 2 2 2 1
HOXA11 level high low
p=2.925e-01
high 186 131 76 49 30 17 11 5 3 1 1 1 1 1 1 0
low 187 140 87 51 33 17 10 9 6 5 4 2 1 1 1 1
HOXA13 level high low
p=4.592e-01
high 186 139 80 53 35 17 12 7 5 3 3 1 1 1 1 0
low 187 132 83 47 28 17 9 7 4 3 2 2 1 1 1 1
HOXB1 level high low
p=2.727e-01
high 186 143 82 53 32 23 14 10 7 4 3 2 1 1 1 1
low 187 128 81 47 31 11 7 4 2 2 2 1 1 1 1 0
HOXB2 level high low
p=7.712e-01
high 186 132 81 48 34 21 12 8 5 3 3 1 1 1 1 0
low 187 139 82 52 29 13 9 6 4 3 2 2 1 1 1 1
HOXB3 level high low
p=6.709e-01
high 186 135 83 47 31 15 8 4 4 2 2 1 1 1 1 1
low 187 136 80 53 32 19 13 10 5 4 3 2 1 1 1 0
HOXB4 level high low
p=5.117e-01
high 186 135 74 48 31 17 9 6 4 2 2 0 0 0 0 0
low 187 136 89 52 32 17 12 8 5 4 3 3 2 2 2 1
Disease free survival probability
Time(years)

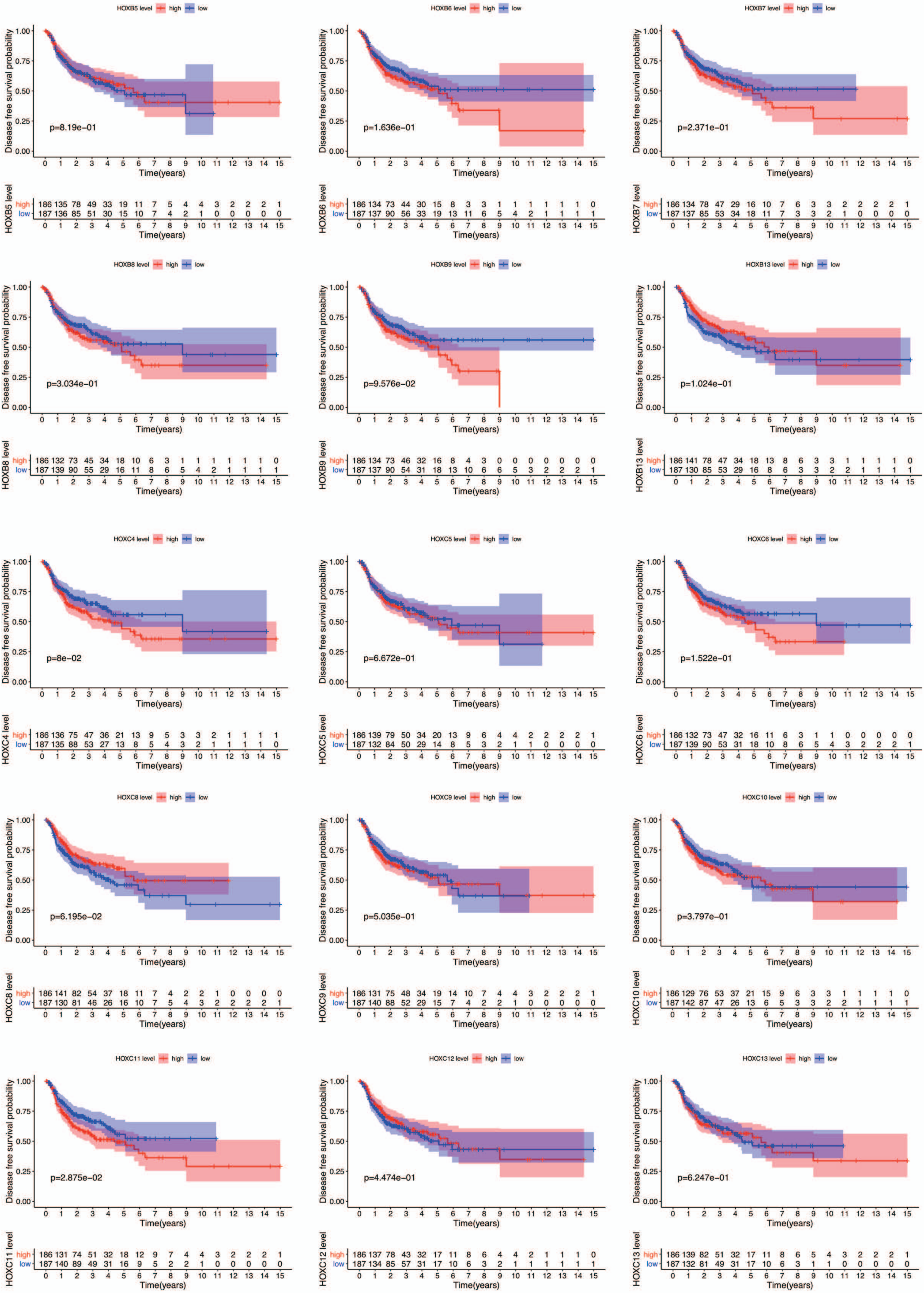
HOXB5 level high low
Disease free survival probability
Time(years)
p=8.19e-01
high 186 135 78 49 33 19 11 7 5 4 4 3 2 2 2 1
low 187 136 85 51 30 15 10 7 4 2 1 0 0 0 0 0
HOXB6 level high low
p=1.636e-01
high 186 134 73 44 30 15 8 3 3 1 1 1 1 1 1 0
low 187 137 90 56 33 19 13 11 6 5 4 2 1 1 1 1
HOXB7 level high low
p=2.371e-01
high 186 134 78 47 29 16 10 7 6 3 3 2 2 2 2 1
low 187 137 85 53 34 18 11 7 3 3 2 1 0 0 0 0
HOXB8 level high low
p=3.034e-01
high 186 132 73 45 34 18 10 6 3 1 1 1 1 1 1 0
low 187 139 90 55 29 16 11 8 6 5 4 2 1 1 1 1
HOXB9 level high low
p=9.576e-02
high 186 134 73 46 32 16 8 4 3 0 0 0 0 0 0 0
low 187 137 90 54 31 18 13 10 6 6 5 3 2 2 2 1
HOXB13 level high low
p=1.024e-01
high 186 141 78 47 34 18 13 8 6 3 3 1 1 1 1 0
low 187 130 85 53 29 16 8 6 3 3 2 2 1 1 1 1
HOXC4 level high low
p=8e-02
high 186 136 75 47 36 21 13 9 5 3 3 2 1 1 1 1
low 187 135 88 53 27 13 8 5 4 3 2 1 1 1 1 0
HOXC5 level high low
p=6.672e-01
high 186 139 79 50 34 20 13 9 6 4 4 2 2 2 2 1
low 187 132 84 50 29 14 8 5 3 2 1 1 0 0 0 0
HOXC6 level high low
p=1.522e-01
high 186 132 73 47 32 16 11 6 3 1 1 0 0 0 0 0
low 187 139 90 53 31 18 10 8 6 5 4 3 2 2 2 1
HOXC8 level high low
p=6.195e-02
high 186 141 82 54 37 18 11 7 4 2 2 1 0 0 0 0
low 187 130 81 46 26 16 10 7 5 4 3 2 2 2 2 1
HOXC9 level high low
p=5.035e-01
high 186 131 75 48 34 19 14 10 7 4 4 3 2 2 2 1
low 187 140 88 52 29 15 7 4 2 2 1 0 0 0 0 0
HOXC10 level high low
p=3.797e-01
high 186 129 76 53 37 21 15 9 6 3 3 1 1 1 1 0
low 187 142 87 47 26 13 6 5 3 3 2 2 1 1 1 1
HOXC11 level high low
p=2.875e-02
high 186 131 74 51 32 18 12 9 7 4 4 3 2 2 2 1
low 187 140 89 49 31 16 9 5 2 2 1 0 0 0 0 0
HOXC12 level high low
p=4.474e-01
high 186 137 78 43 32 17 11 8 6 4 4 2 1 1 1 0
low 187 134 85 57 31 17 10 6 3 2 1 1 1 1 1 1
HOXC13 level high low
p=6.247e-01
high 186 139 82 51 32 17 11 8 6 5 4 3 2 2 2 1
low 187 132 81 49 31 17 10 6 3 1 1 0 0 0 0 0

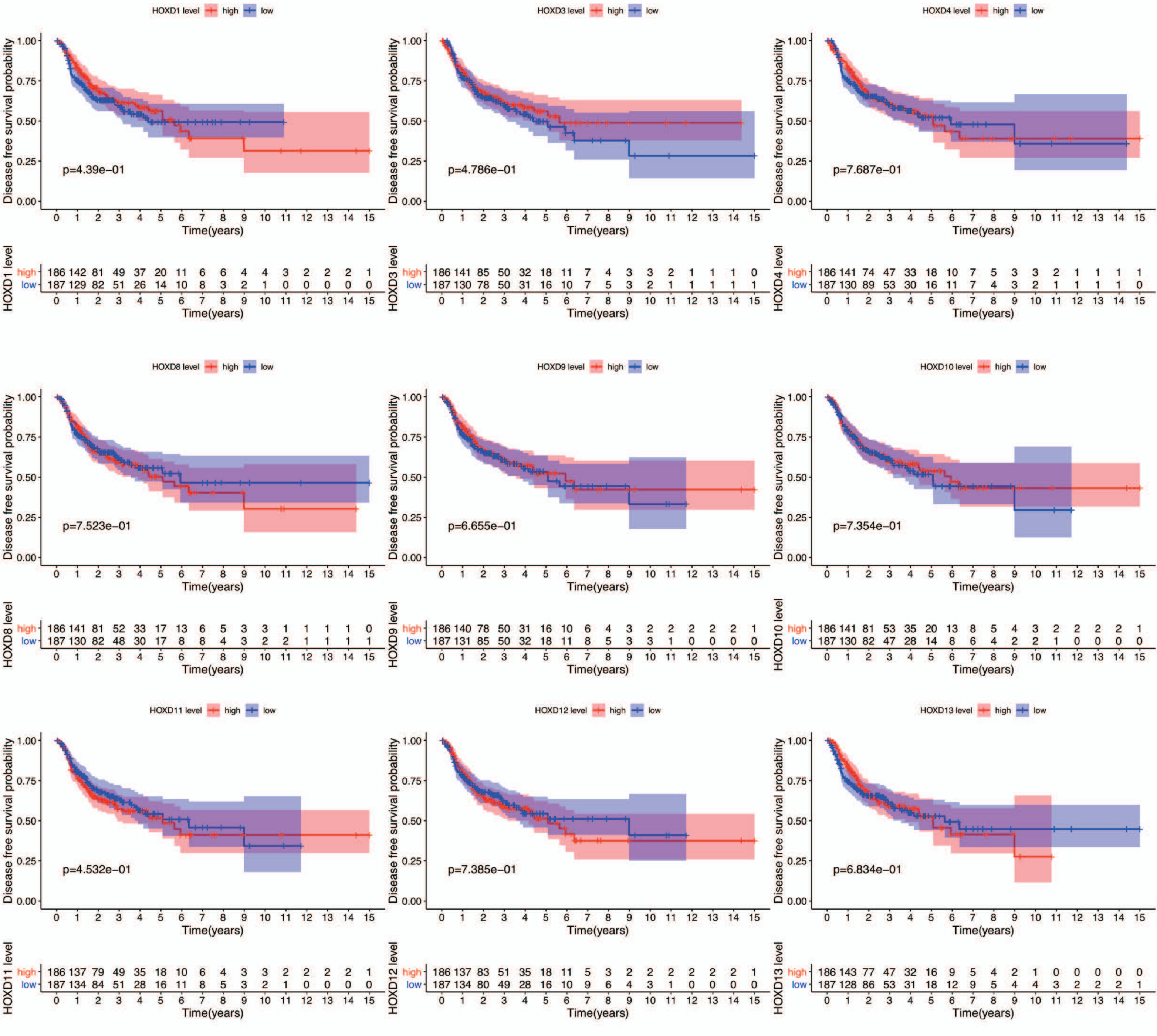

HOXD1 level high low
Disease free survival probability
Time(years)
p=4.39e−01
HOXD1 level
high 186 142 81 49 37 20 11 6 6 4 4 3 2 2 2 1
low 187 129 82 51 26 14 10 8 3 2 1 0 0 0 0 0
HOXD3 level high low
p=4.786e−01
HOXD3 level
high 186 141 85 50 32 18 11 7 4 3 3 2 1 1 1 0
low 187 130 78 50 31 16 10 7 5 3 2 1 1 1 1 1
HOXD4 level high low
p=7.687e−01
HOXD4 level
high 186 141 74 47 33 18 10 7 5 3 3 2 1 1 1 1
low 187 130 89 53 30 16 11 7 4 3 2 1 1 1 1 0
HOXD8 level high low
p=7.523e−01
HOXD8 level
high 186 141 81 52 33 17 13 6 5 3 3 1 1 1 1 0
low 187 130 82 48 30 17 8 8 4 3 2 2 1 1 1 1
HOXD9 level high low
p=6.655e−01
HOXD9 level
high 186 140 78 50 31 16 10 6 4 3 2 2 2 2 2 1
low 187 131 85 50 32 18 11 8 5 3 3 1 0 0 0 0
HOXD10 level high low
p=7.354e−01
HOXD10 level
high 186 141 81 53 35 20 13 8 5 4 3 2 2 2 2 1
low 187 130 82 47 28 14 8 6 4 2 2 1 0 0 0 0
HOXD11 level high low
p=4.532e−01
HOXD11 level
high 186 137 79 49 35 18 10 6 4 3 3 2 2 2 2 1
low 187 134 84 51 28 16 11 8 5 3 2 1 0 0 0 0
HOXD12 level high low
p=7.385e−01
HOXD12 level
high 186 137 83 51 35 18 11 5 3 2 2 2 2 2 2 1
low 187 134 80 49 28 16 10 9 6 4 3 1 0 0 0 0
HOXD13 level high low
p=6.834e−01
HOXD13 level
high 186 143 77 47 32 16 9 5 4 2 1 0 0 0 0 0
low 187 128 86 53 31 18 12 9 5 4 4 3 2 2 2 1
